# Data on Vietnamese patients׳ financial burdens and risk of destitution

**DOI:** 10.1016/j.dib.2016.09.040

**Published:** 2016-09-30

**Authors:** Quan-Hoang Vuong, Trong-Khang Nguyen

**Affiliations:** a FPT School of Business (FSB), FPT University, VAS-FSB Building, Block C, My Dinh 1, Tu Liem District, Hanoi, Vietnam; b MK Group (Vietnam), 11th Floor, TTC Building, Duy Tan, Cau Giay District, Hanoi, Vietnam

## Abstract

The research process started in the first week of August 10, 2014 and ended early February 2015, obtaining qualified data for 330 patients from many hospitals in northern Vietnam. Its expansion was performed for an enlarged dataset through May 2015, containing 900 records. This article exemplifies the attempt to examine the likelihood of destitution among Vietnamese patients due to insufficient insurance coverage, cost of treatment and patient׳s status of residency during a curative hospital stay. The result suggests that the patients, who are poor and come from rural areas, face serious obstacles in accessing health care services. This data article presents attributes and values of the data set used in the article provided at DOI: http://dx.doi.org/10.1186/s40064-015-1279-x Vuong (2015) [4].

**Specifications Table**

**Table:** 

Subject area	*Medical*
More specific subject area	*Patient׳s financial burdens occurring during a curative hospital stay*
Type of data	*Table, graph, figure*
How data was acquired	*Survey*
Data format	*Raw, filtered, and partially analyzed*
Experimental factors	*Raw data obtained from a survey patients at many hospitals in Northern Vietnam*
Experimental features	*The experiment focuses on examining financial issues, illness, insurance, end result of treatment, health care costs, length of stay, ‘envelope OOP’ and the probability of post-treatment destitution, for different groups of patients*
Data source locations	*Viet Duc, Bach Mai, Vietnam-Japan, Hai Duong Polyclinic, Thai Binh Polyclinic Hospitals, Vietnam (and others)*
Data accessibility	*Both original datasets for 330 and 900 survey records are provided with this article, and deposited at DOI:*http://doi.org/10.17632/rw28hh58y4.1.

**Value of the data**

• The data potentially offer a specific insight on financial destitution due to costs of treatment.
• The data can help reveal the likelihood of patient׳s recovery after treatment.
• The data help to understand the effect of health insurance on reducing the likelihood of falling into destitution.
• The data shed light on the risk of “extra thank-you money” becoming an obligatory part of treatment costs for patients, low-income patients in particular.
• The data set structure can be enriched for deeper analysis providing insights and implications for bettering policy making in health insurance and poverty reduction.

## 1 Data

**Fig. 1:**
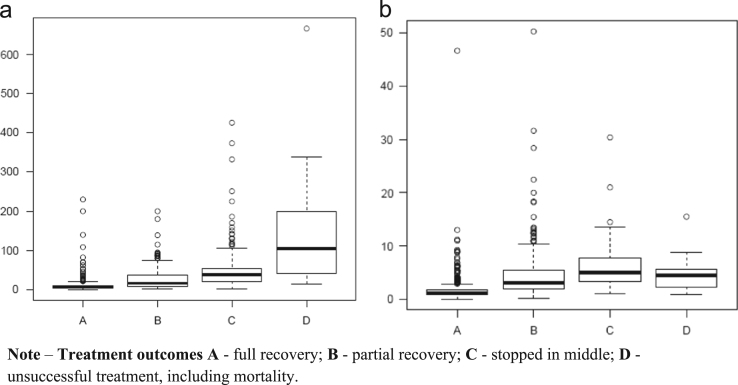
Status of health in relation to expenditure and average daily cost. (a) Range of expenses for patients with treatment outcome categories (A, B, C or D). (b) Range of daily cost for patients falling into different treatment outcome groups (A, B, C or D).

The first data set, which will be used for computation examples provided in this article, contains 900 records obtained from a survey on Vietnamese inpatients concerning family status, patient׳s income level, patient׳s extra expenses to doctors and hospital׳s staff, and their loans to finance treatment. Fig. 1 shows the distribution of patients hospitalization lengths.

Continuous (numerical) and discrete (categorical) variables are measured and reported in the survey data set. Table 1 presents numerical variables of the data set. Fig. 1a and b present medical expenditures and average daily costs in relation to health status of patients when hospitalized. They are not significantly different from those provided in [4] (Table 2).

## 2 Experimental design, materials and methods

The dataset was constructed using information from questionnaires in order to enable the modeling of baseline-category logits (BCL). In addition, the computing of empirical probabilities upon events of hypothetical influence is performed. The logic for designing the experiment and thus data sets are similar to what is described in [1], for data groups in J categories of Y as multinomial with corresponding sets of probabilities $\left\{\pi_{1}\left(x\right),\ldots ,\pi_{j}\left(x\right)\right\}$, and with the multinomial probability mass function: $p\left(n_{1},n_{2},\ldots ,n_{c}\right)=\left(\frac{n!}{n_{1}!n_{2}!\ldots n_{c}!}\right)\pi_{1}^{n_{1}}\pi_{2}^{n_{2}}{\ldots \pi }_{c}^{n_{c}}.$ The data set has been created to enable an BCL analysis to simultaneously model effects of x on (J−1) logits such that the estimating of (J−1) equations enables the computing of the remaining logits. Therefore, Pearson-type likelihood ratio test statistics $X^{2},G^{2}$ or goodness-of-fit, following a multivariate GLM estimations $g\left(\mu_{i}\right)=X_{i}\beta$ become appropriate for hypothesis testing. For practically estimating multinomial logistic models, consult with Refs. [2], [3]. Practicality of survey data uses is also provided in Ref. [4].

Some possible questions and hypotheses worth testing of, using the data set analyzed by [4], is in Table 3.

The following short R commands help create the data set provided in the file named “table1.csv”

**Table:** 

>med=read.csv(“E:/…/Med2015/Data/P330.csv,header=T)
>attach(med)
>table1=xtabs(~Res+Insured+Burden)
>ftable(table1)

The data set in file name “table1.csv” presents a distribution of patients following by residency status, insurance participation, and extent to which patients fall into destitution due to financial hardships after treatment. Its modeling using the BCL method is performed by R commands as follows:

**Table:** 

> burden1=read.csv(“E:/…/Med2015/Data/table1.csv,header=T)
> attach(burden1)
> contrasts(burden1$Res)=contr.treatment(levels(burden1$Res),base=2)
> contrasts(burden1$Insured)=contr.treatment(levels(burden1$Insured),base=2)
> fit.burden1=vglm(cbind(C,B,A)~Res+Insured,family=multinomial,data=burden1)
> summary(fit.burden1)

**Fig. 2:**
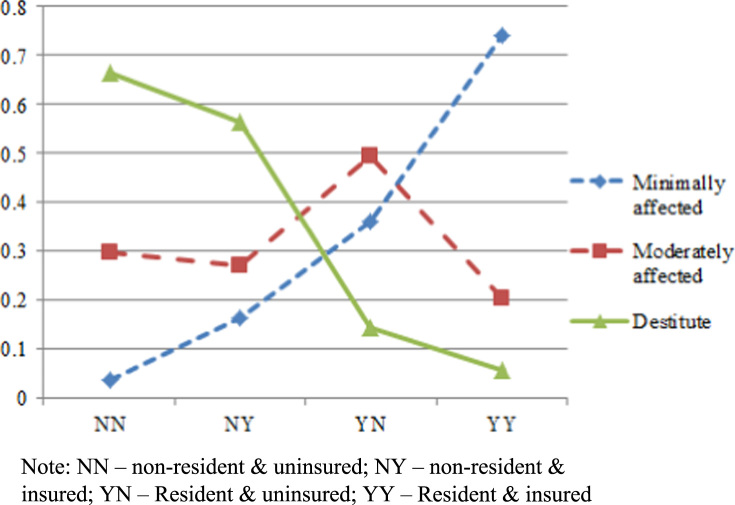
Contrasting financial welfare of patients as a function of status of residency and insurance cover/lack of cover.

The above estimation yields coefficients and associated statistics that are used in [4] for estimating empirical probabilities, using the 330-observation dataset. In addition, Fig. 2 is drawn for the 900-observation dataset.

The probabilistic trends for patient׳s financial burdens are in line with [5]. Examples of R commands for creating a specific dataset and corresponding BCL estimations are provided in Appendixes A and B, Appendixes A and B.

**Fig. 3:**
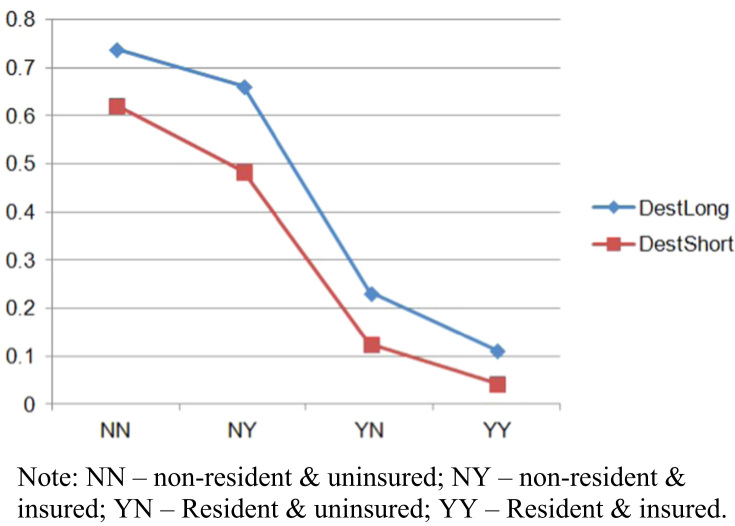
Changing probabilities of destitution for patients as a function of short versus long hospitalization.

In the same vein, Fig. 3 shows declining trends for becoming destitute if a patients is either resident or insured, or both.

## Figures and Tables

**Table:** 

>med=read.csv(“E:/…/P330.csv,header=T)
>attach(med)
> table2=xtabs(~AvgCost+Insured+Burden)
> ftable(table2)

**Table:** 

> burden2=read.csv(“E:/…/table2.csv,header=T)
> attach(burden2)
> contrasts(burden2$AvgCost)=contr.treatment(levels(burden2$AvgCost),base=2)
> contrasts(burden2$Insured)=contr.treatment(levels(burden2$Insured),base=2)
> fit.burden2=vglm(cbind(C,B,A)~AvgCost+Insured,family=multinomial,data=burden2)
> summary(fit.burden2)

**Table 1:** Numerical variables of the data set.

Coded name	Explanation	Unit
Spent	Total money spent during his/her stay in hospital in millions of Vietnamese Dong. According to official exchange rates at the time of survey, VND 1 million was equivalent to $47.2.	Million of VND
Dcost	Average daily cost the patient had to pay during the entire treatment period	Million of VND
Income	Annual income	Million of VND
Days	Number of days the patient spent in the hospital	Day
Pins; Pinc; Pchar; Ploan	Portions of finance from sources: insurance reimbursement, income, charity funds from civil organizations or employers, or borrowings	Percent
Streat, Srel, Senv	Percentage of funds used for the purpose of main treatments, for covering costs of relatives coming to help the patient, or paying “extra thank-you money” or bribing doctors/staff	Percent

**Table 2:** Categorical variables of the data set.

Coded name	Explanation	Values
Res	Whether the patient originally resides in the region where the hospital is located	Yes, no
Stay	A dummy variable to define the patient׳s hospitalization by length of stay	Less than 10 days (S), 10 days or longer (L)
Insured	Whether a patient has a valid health insurance	Yes, no
SES	Socio-economic status	High, medium, low
		Based on average IncRank of working members in the family who are able and willing to support the patient if so required
Illness	Severity of illness or injury when hospitalized	Emergency, bad, ill, light
IncRank	Rankings of income of a patient	High (>180), middle (48–180), low (<48)
		Excel:
		=IF([cell]>180,"Hi",IF([cell]>48,"Mid","Lo"))
Burden	Patient׳s and family׳s self-evaluation of their financial position after paying health care costs	strong, no adverse affect at all (A); affected but not the worrying level (B); seriously affected or destitute/bankrupt (C)
End	Patient׳s health status after treatment	complete recovery (A), partial recovery, needing post-treatment follow-ups (B), stopped whilst being treated (C), or quit early (D)
AvgCost	Average daily cost the patient had to pay during treatment period	≤1.5 (Low), 1.5 to 5.4 (Med), and >5.4 (Hi)

**Table 3:** Possible research questions arising from the data set.

Do the residency status of patients and insurance coverage determine the probability of patients falling into debts? The specific factor of residency status is important in Vietnam because society has for long been skeptical about provincial healthcare, leading patients to travel to large hospitals in major cities such as Hanoi, Hai Phong, or HCMC. Doing so not only necessitates accompanying and caretaking of family members but also entails travel costs and informational asymmetry on drug prices, treatment schedules, the best hospital to visit and even the ‘right amount’ of “extra thank-you money” (a kind of out-of-pocket expense; or OOP).

As for two most important factors to Vietnamese patients/households, i.e. treatment costs and illness, is there evidence to support this view and if yes, whose influence better explains the possibility of end results of treatment, empirically?

Can the likelihood of paying too little or too much out-of-pocket “extra thank-you money” be determined by the severity of illness and/or income of patients? This OOP amount may be significant but if a patient appreciates the value of service, he/she would be willing to pay depending on his/her availability of finance, before or after the course of treatment.

## Appendix A.

R commands help create the data set provided in the file named “table2.csv”

## Appendix B.

R commands using data in “table2.csv” estimated model follow as:
