# Supplementary material for: Data on Vietnamese patients׳ financial burdens and risk of destitution
Source: Data Brief. 2016 Sep 30;9:543–8. doi: 10.1016/j.dib.2016.09.040 (PMC5061292; doi:10.1016/j.dib.2016.09.040)
Supplement: Supplementary file 2 — Supplementary material [file mmc1.pdf]

The Statement on Conflict of Interest

I the undersigned and corresponding author of the manuscript:

Data on Vietnamese patients' financial burdens and risk of destitution

hereby state that we the authors have not conflict of interests, now or potentially, with the survey and the dataset and the publishing process of this data article.

We have received no funding from any institutions.

The sole purpose of this data survey process is to make a serious research attempt into the topic that the article describes.

We will take all responsibilities for any misstatement or wrong declaration in this.

On behalf of co-authors

Quan Hoang Vuong, Ph.D.
FPT University, School of Business
hoangvq@fsb.edu.vn

Quan Hoang Vuong

Digitally signed by Quan Hoang Vuong
DN: cn=Quan Hoang Vuong, o=Universite Libre de Bruxelles, ou=Centre Emile Bernheim, email=qvuong@ulb.ac.be, c=BE
Date: 2016.08.30 14:46:47 +07'00'
